# Minimal access surgery for congenital diaphragmatic hernia: surgical tricks to facilitate anchoring the patches to the ribs

**DOI:** 10.1007/s00383-022-05303-y

**Published:** 2023-02-20

**Authors:** Sherif Mansour, Joe Curry, Simon Blackburn, Dhanya Mullassery, Hemanshoo Thakkar, Jennifer Ballington, Stavros Leukogeorgakis, Kate Cross, Stefano Giuliani, Paolo De Coppi

**Affiliations:** 1 Great Ormond Street Hospital for Children, Surgery Offices, Zayed Centre for Research, UCL Institute of Child Health, 30 Guilford Street, London, WC1N 1EH UK; 2 Present address: Evelina Children’s Hospital, London, UK

**Keywords:** Congenital, Diaphragmatic hernia, Patch, Thoracoscopic, Endo close

## Abstract

**Objective:**

Minimal Access Surgery (MAS) for Congenital Diaphragmatic Hernia (CDH) repair is well described, yet only a minority of surgeons report this as their preferred operative approach. Some surgeons find it particularly difficult to repair the defect using MAS and convert to laparotomy when a patch is required. We present in this study our institutional experience in using an easy and relatively cheap methodology to anchor the patch around the ribs using Endo Close™. This device has an application in MAS for tissue approximation using percutaneous suturing.

**Methods and technique:**

We retrospectively reviewed our database for patients undergoing MAS repair of CDH between 2009 and 2021. Outcome measures included length of surgery and recurrence rates after patch repair. Endo Close™ was used in all patients who required patch repair. We declare no conflict of interest and to not having received any funding from Medtronic (UK). The technique is as follows: (1) The edges of the diaphragm are delineated by dissection. When primary suture repair of the diaphragmatic hernia was unfeasible without tension, a patch was used. (2) The patch is anchored in place by two corner stitches at the medial and lateral borders. (3) The posterior border of the patch is fixed to the diaphragmatic edge by running or interrupted stitches. (4) For securing the anterior border, a non-absorbable suture is passed through the anterior chest wall and the patch border is taken with intracorporeal instruments. (5) Without making another stab incision, the Endo Close™ is tunnelled subcutaneously through the anterior chest wall. (6) The suture end is pulled through the Endo Close™ and the knot is tied around the rib. This procedure can be performed as many times as required to secure the patch.

**Results:**

58 patients underwent MAS surgery for repair of CDH between 2009 and 2021. 48 (82%) presented with a left defect. 34 (58%) had a patch repair. The length of patch repair surgery for CDH ranged from 100–343 min (median 197). There was only one patient (3%) in the patch repair cohort that had a recurrent hernia, diagnosed 12 months after the initial surgery.

**Conclusions:**

In our experience, MAS repair of CDH is feasible. We adopted a low threshold in using a patch to achieve a tension-free repair. We believe that the Endo Close™ is a cheap and safe method to help securing the patch around the ribs.

## Introduction

Congenital diaphragmatic (CDH) occurs in approximately 1 in 2500–4000 live births [1]. It results from failure of complete fusion of the developing foetal diaphragm—a process that normally occurs between gestational weeks 6 and 8. Migration of abdominal organs into the thoracic cavity, pulmonary hypoplasia and respiratory failure at birth can occur.

Current management of CDH in neonates usually involves initial ventilatory support and supportive care, to allow labile cardiopulmonary physiology to improve, followed by surgical reduction of the hernia, usually through an abdominal approach, and repair of the diaphragmatic defect. Gentle ventilation strategies, high frequency oscillatory ventilation (HFOV), extracorporeal membrane oxygenation (ECMO), and other methods of supportive care have changed the intensive care management of CDH [2].

Such advancement it the care of these new-borns has significantly improved the overall prognosis of this previously considered as a lethal anomaly. This has allowed some paediatric surgical centres around the world to shift their practice from the classic repair of CDH through thoracotomy or laparotomy to using minimally invasive surgery.

Throughout the last 2 decades, we have adopted the thoracoscopic approach for the CDH repair in our institute. While initially we have used high CO_2_, we have more commonly adopted a policy of maintaining pressure between 4–6 mmHg. The procedure is mostly performed by a registrar or senior fellow, always supervised by a consultant assisting. We have maintained the same criteria than open repair with a very low threshold for patch repair to decrease the tension and avoid recurrence. We have worked on some technical aspects of the procedure to make it less challenging and to decrease the operative time, while maintaining and even improving our surgical outcomes.

**Fig. 1:**
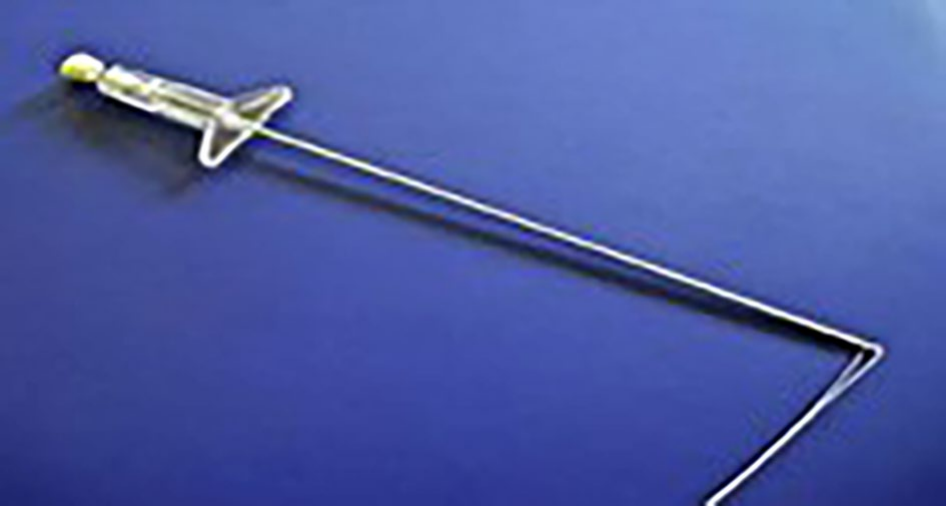
Endo Close™ device

We present in this study our institutional experience in using an easy and relatively cheap methodology to anchor the patch around the ribs using Endo Close™ (Fig. 1). This device has an application in MAS for tissue approximation using percutaneous suturing.

## Materials and methods

### Patients

A retrospective review of all CDH patients undergoing thoracoscopic patch repair at Great Ormond Street Hospital. The study period was between 2009 and 2021. Patients’ data was obtained from our electronic database. Data was also obtained from an audit that was carried out at our institute. The audit was a comparison between thoracoscopic and open repair of congenital diaphragmatic hernia (Audit number #3126). Patients were excluded from the study if their hernia was recurrent or if their hernia was preoperatively diagnosed as a Morgagni hernia. We recently included in our study the patients that needed ECMO support preoperatively. Primary outcome measures included length of surgery and recurrence rates after patch repair. Endo Close™ was used in all patients who required patch repair. We declare no conflict of interest and to not having received any funding from Medtronic (UK).

### Operative technique

Thoracoscopic repair of CDH in neonates is carried out with the patient under general anaesthesia and in the lateral decubitus position. We usually use 3 to 4 trocars including the camera port, with CO_2_ insufflation of the pleural space to partially collapse the lung sufficiently to achieve good exposure of the defect and to reduce the herniated viscera within the abdomen.

The operative technique is as follows: (Fig. 5)

**Fig. 2:**
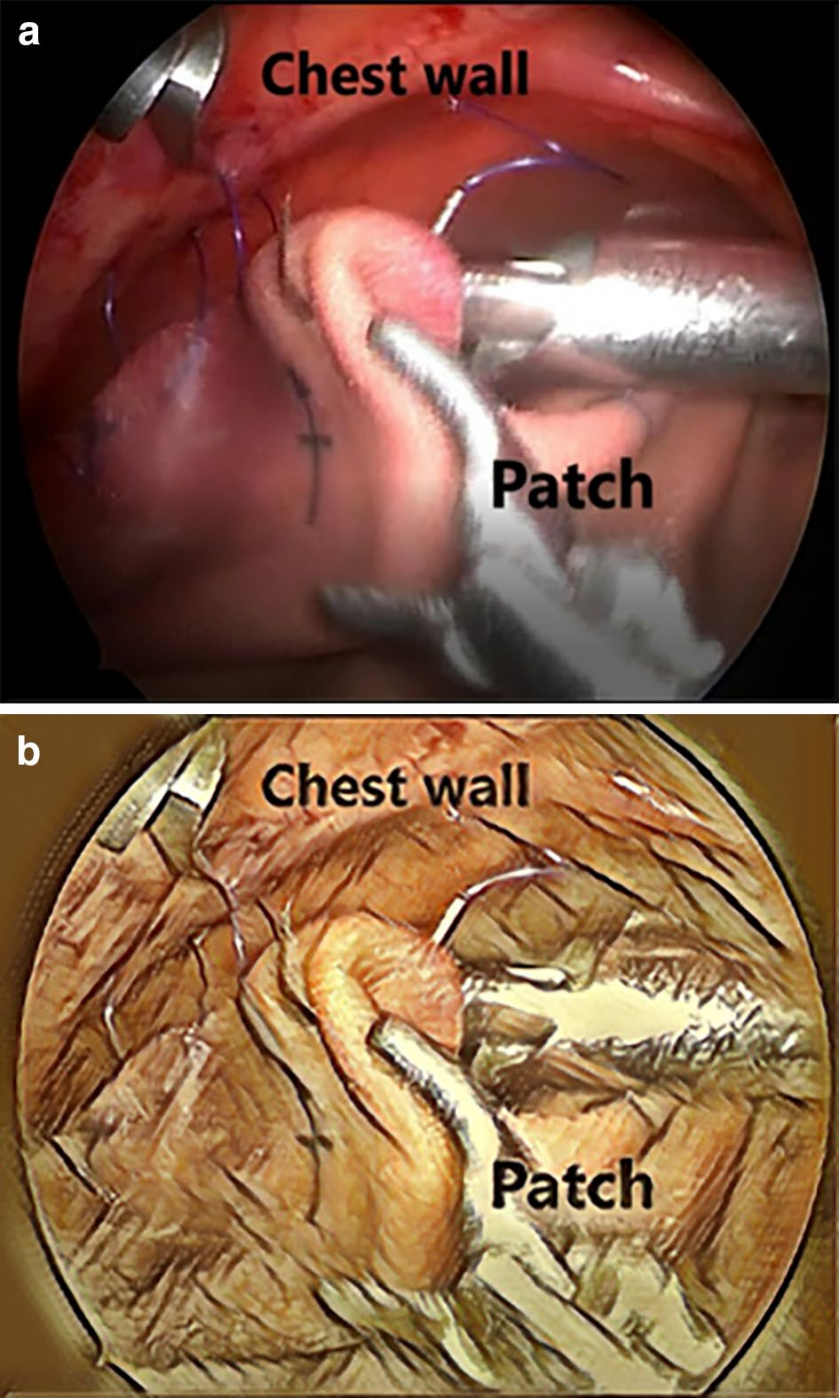
A non-absorbable suture is passed through the chest wall and then through the patch

**Fig. 3:**
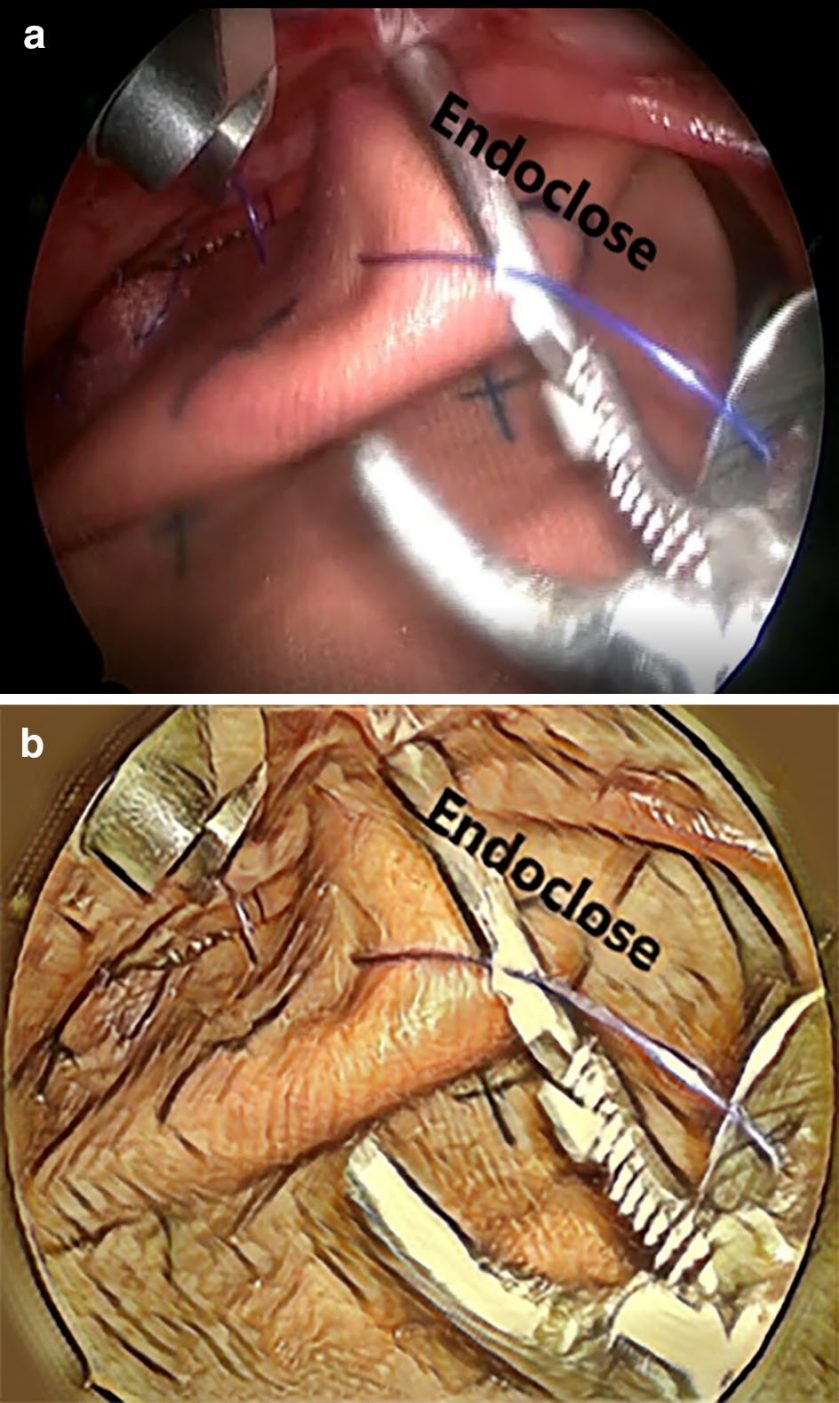
The Endo Close™ is tunnelled through the chest wall and into the chest cavity

**Fig. 4:**
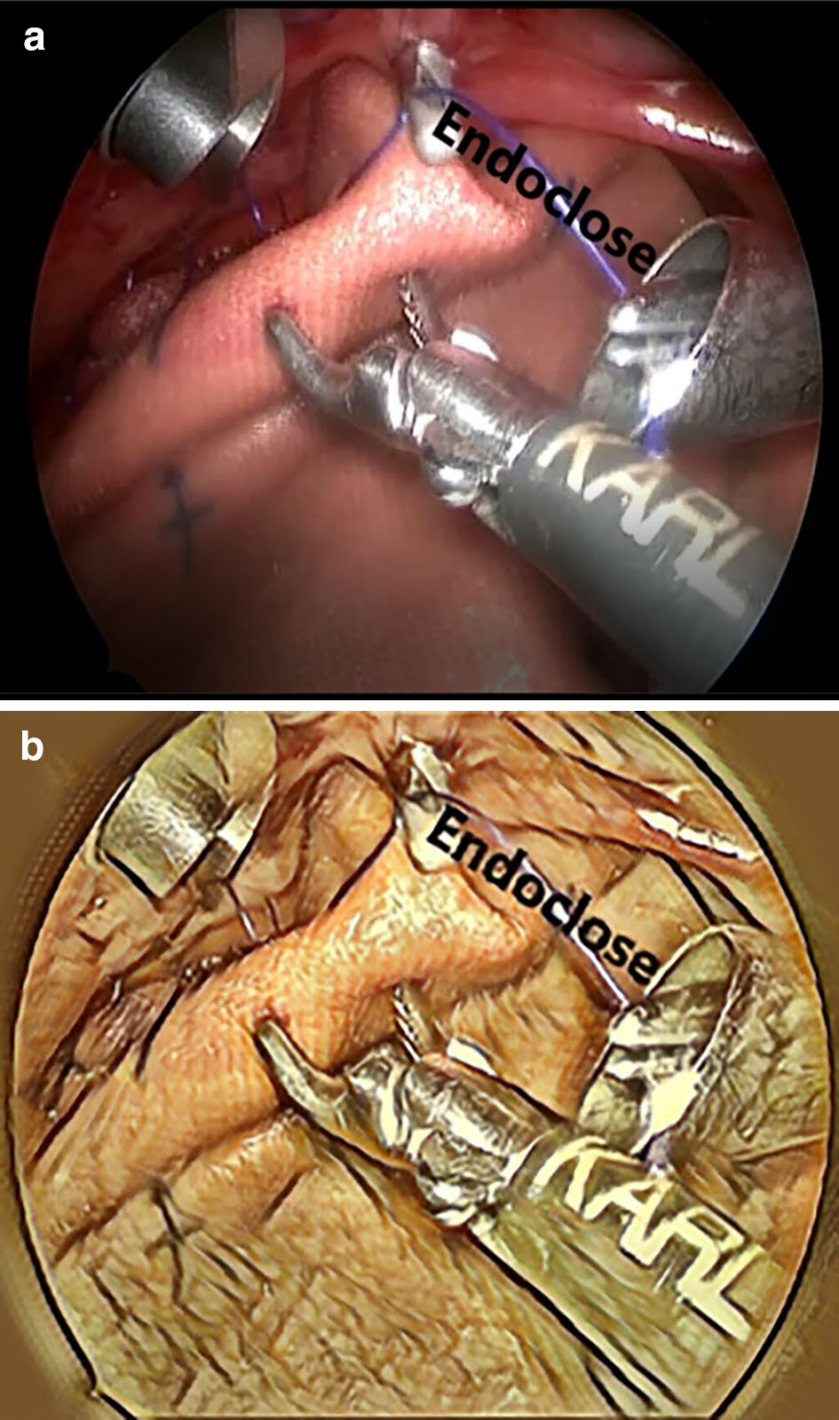
The suture end is pulled through the chest wall with the use of Endo Close™

1. The herniated viscera are reduced gently into the abdominal cavity until the edges of the diaphragm can be visualised. At that stage the CO_2_ pressure is increased to 6 mmHg.
2. The edges of the diaphragm are delineated by dissection. When primary suture repair of the diaphragmatic hernia was unfeasible without tension, a patch was used.
3. The patch is anchored in place by 2 corner stitches at the medial and lateral borders of the diaphragmatic edges.
4. The posterior border of the patch is fixed to the diaphragmatic edge by running or interrupted stitches. Of note, most CDH repairs were done by running stitches (usually 2), which helps to decrease the operative time.
5. For securing the anterior border, a non-absorbable suture is passed through the anterior chest wall through a small stab skin incision. The suture is then passed through the anterior patch border with intracorporeal instruments (Fig. 2).
6. Without making another stab incision, the Endo Close™ is tunnelled subcutaneously through the anterior chest wall (Fig. 3).
7. The suture end is pulled through the Endo Close™ and the knot is tied around the rib in the subcutaneous space. This procedure can be performed as many times as required to secure the patch. The same skin incision can be used to secure 2–3 stitches before needing to do another separate incision (Fig. 4).

### Statistical analysis

Descriptive data included side of defect. Operative characteristics included operative time and intra-operative complications. Post-operative variables included recurrence and complications.

Descriptive statistics for measured variables will be expressed as range, mean and standard deviation (for metric data); range, median and interquartile range (for discrete data); and number and proportions (for categorical data).

## Results

All patients were born at an outside institution and transferred to Great Ormond Street Hospital. 58 patients underwent thoracoscopic repair of CDH between 2009 and 2021. 48 (82%) presented with a left defect.

The study group included 34 patients, all of whom underwent MAS patch repair. Endo Close™ was used in all the patients. Size of the defect of the patients’ cohort ranged from 3 × 4 cms to 12 × 10 cms (Median 4 × 5 cms). All the study group patients have been followed up until the time of writing of this paper.

The operative time ranged from 100–343 min (Median 197). There were 2 intra-operative complications (5.8%). One patient developed supra-ventricular tachycardia (SVT) intra-operatively requiring DC cardioversion. Another patient had extravasation of noradrenaline and dopamine.

While 3/58 (5%) patients done thoracoscopically had a recurrence, 1/34 (3%) patients in the study group had a recurrent hernia. Post-operative complications occurred in 7 (20%) patients. Complications included 3 patients who developed post-operative pneumothorax, 2 of them needed a chest drain. Laparoscopy was done in one patient for suspected bowel obstruction. One patient had closure of chest drain site under general anaesthesia. One patient developed chylothorax that was managed conservatively. Two patients developed post-operative wound infection. [Table 1].

**Table 1:** Pre, intra and post-operative characteristics of CDH patients who underwent thoracoscopic patch repair

Measured variable	Study Group n = 34
Size of defect	3 × 4 cms to 12 × 10 cms (Median 4 × 5cms)
Intra-operative variables	
Operative time	100–343 min (Median 197)
Intra-operative complications	2 (5.8%)
	1. 1 patient developed SVT
	2. 1 patient had extravasation of noradrenaline and dopamine
Post-operative variables	
Recurrence	1 (2.9%)
Post-operative complications	7 (20%)
3. Pneumothorax	3
4. Chylothorax	1
5. Suspected bowel obstruction	1
6. Closure of chest drain site under GA	1
7. Wound infection	2

## Discussion

Over the past few years, there has been a significant progress in the management of CDH patients. This was reflected by the improved prognosis and decreased morbidity and mortality of these patients. Alongside, there has been a growing interest in thoracoscopic repair of CDH. Thoracoscopy has been suggested to have significant advantages over the traditional open approaches. These benefits include a decrease in the pain and incisional morbidity of a thoracotomy (i.e., subsequent scoliosis, chest deformities, and shoulder muscle girdle weakness), reduced surgical stress and immunologic derangement, faster recovery, and shorter hospitalizations [3]. Historically, the patch repair of CDH has been associated with a higher rate of recurrence and complications. In the last decade, few publications have concluded results that questioned that common belief.

In our institute, we have been adopting a low threshold for patch repair of CDH to achieve a tension-free repair. Based on a cohort study that was carried at Great Ormond Street Hospital, patch repair of CDH was not associated with higher recurrence, though access route was. The study included 203 patients, 107 received a patch, and 96 were not patched. Groups were not different for gestational age birthweight, gender, defect side and minimally invasive approach rate. Preoperative ECMO incidence (*p*:29.9% vs. NP:2.1%, *p* < 0.01), liver herniation (*p*:57.0% vs. NP:22.9%, *p* < 0.01) and absence of a postero-lateral rim (P:61.7% vs. NP:8.3%, p < 0.01) were higher in the P group. The mortality rate was 10.8% (*p*:15.0% vs. NP:6.2%, *p* = 0.07). Recurrence was not different (*p*:9.3% vs. NP:4.2%, *p* = 0.15). Multivariate analysis showed that recurrence was higher after thoracoscopy compared to open (OR = 12.2 [2.2–68], *p* < 0.01); neither the use of patch (OR = 2.3, [0.5–10.4], *p* = 0.28) nor any other factors were associated with recurrence [4].

Another study demonstrated that synthetic patch repair for CDH had a low rate of recurrence. One hundred eighty-four children underwent CDH repair of whom 99 (53.8%) required a patch. Seventy-four (74.7%) of the 99 patients who underwent patch repair survived to discharge and were compared with 75 primary repair survivors. Two patients experienced a recurrence after a patch repair and 3 after a primary repair for a rate of 5.4 and 4.0%, respectively (*p* = 1.0) [5].

In our current study, only one patient out of the 34 (2.9%) had recurrence after thoracoscopic patch repair of CDH. This reflects that with our growing experience in the technique, the rate of complications is showing a downward trend.

We are continuously seeking to refine our surgical technique to improve our peri-operative variables, including operative time and recurrence rates. We have found that using the Endo Close ™ is a useful and a cheap adjunct that makes the intracorporeal fixing of the patch more technically feasible.

## Conclusion

In our experience, MAS repair of CDH has got comparable results to the open repair. We adopted a low threshold in using a patch to achieve a tension-free repair. We believe that the Endo Close™ is a cheap and safe method to help us to secure the patch.

**Fig. 5:**
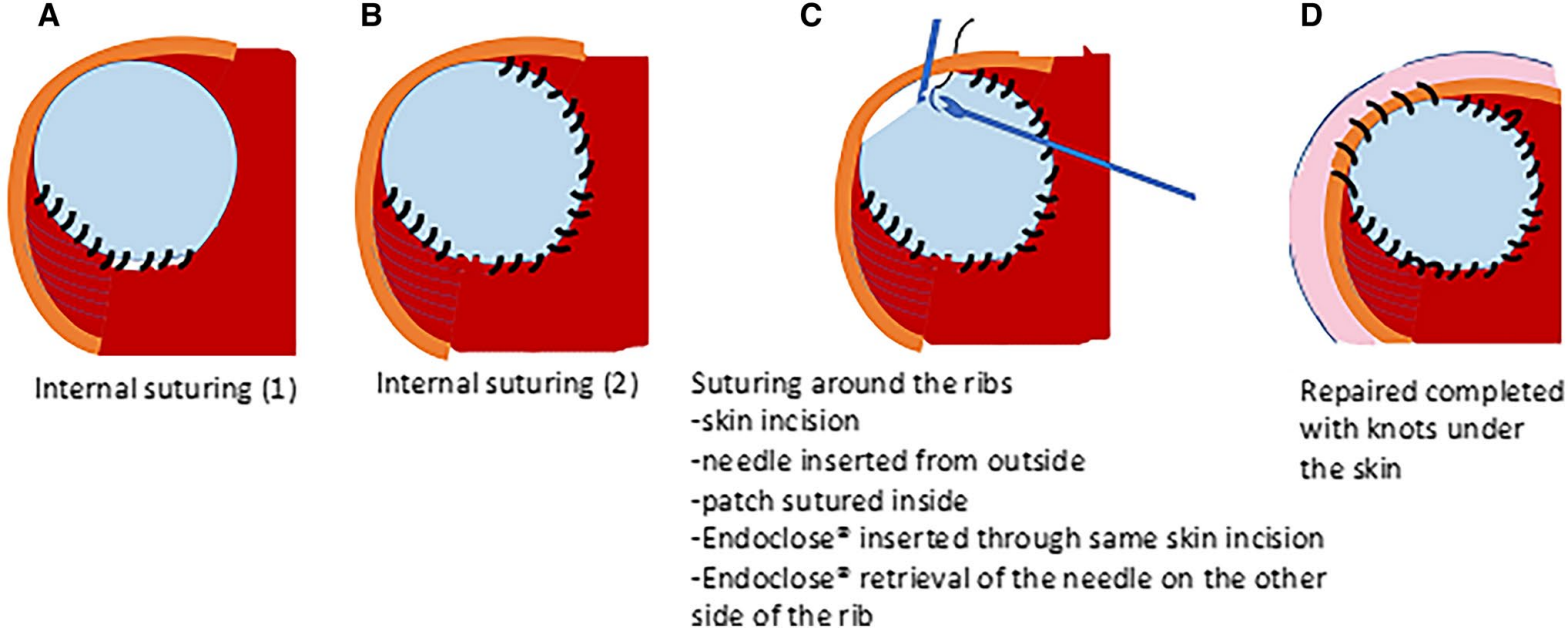
Diagram illustrating the steps of suturing the patch all around until the repair of the hernia is complete

## Data Availability

The authors confirm that the data supporting the findings of this study are available within the article and its supplementary materials.
